# The *I.3.2* developmental mutant has a single nucleotide deletion in the gene *centromere identifier*

**DOI:** 10.17912/micropub.biology.000653

**Published:** 2022-10-25

**Authors:** Cory J. Evans, Kayla L. Bieser, Katherine S. Acevedo-Vasquez, Emyli J. Augustine, Skyler Bowen, Veronica A. Casarez, Vanessa I. Feliciano, Ashley Glazier, Haley R. Guinan, Randy Hallman, Elizabeth Haugan, Lauren A. Hehr, Shawna N. Hunnicutt, Isabella Leifer, Meaghan Mauger, Morgan Mauger, Norma Y. Melendez, Larry Milshteyn, Eric Moore, Sarah A. Nguyen, Sierra C. Phanphouvong, David M. Pinal, Hailee M. Pope, Mark-Brandon M. Salinas, Matthew Shellin, Ivana Small, Neelufar C. Yeoh, Alexandra M.K. Yokomizo, Jacob D. Kagey

**Affiliations:** 1 Department of Biology, Loyola Marymount University, Los Angeles, CA, USA; 2 Department of Physical and Life Sciences, Nevada State College, Henderson, NV, USA; 3 Biology Department, University of Detroit Mercy, Detroit, MI, USA

## Abstract

The mutation *I.3.2* was previously identified in a FLP/FRT screen of chromosome 2R for conditional growth regulators. Here we report the phenotypic characterization and genetic mapping of *I.3.2* by undergraduate students participating in Fly-CURE, a pedagogical program that teaches the science of genetics through a classroom research experience. We find that creation of *I.3.2* cell clones in the developing eye-antennal imaginal disc causes a headless adult phenotype, suggestive of both autonomous and non-autonomous effects on cell growth or viability. We also identify the *I.3.2* mutation as a loss-of-function allele of the gene *centromere identifier* ( *cid* ), which encodes centromere-specific histone H3 variant critical for chromosomal segregation.

**Figure 1. Phenotypic characterization, genetic mapping, and molecular analysis of mutation I.3.2 .**
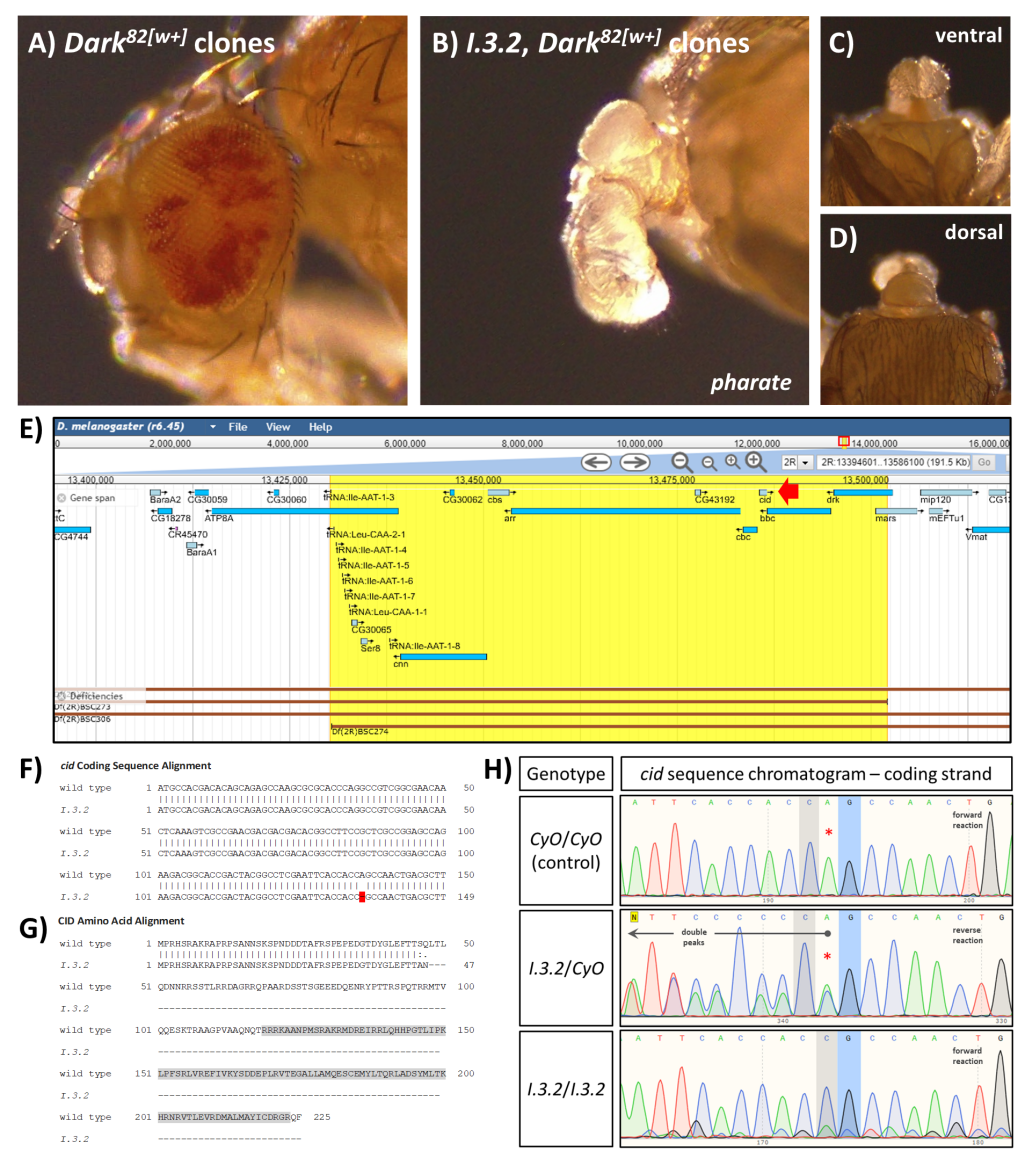
A) Adult fly eye phenotype (control) showing mosaic pigmentation caused by *Dark ^82[w+]^* (red) and wild-type (white) mitotic cell clones; B) Adult “headless” phenotype (dissected pharate) in flies creating *I.3.2, Dark ^82[w+]^* and wild-type mitotic cell clones. Residual structures resemble adult mouthparts and arise from tissues not creating mutant clones. Headless *I.3.2* clone phenotype from ventral (C) and dorsal (D) views. All images are 55X magnification. E) Genomic view of the *cid* locus (FlyBase) showing chromosomal deficiencies (red bars, bottom) that delete *cid* . The highlighted region (yellow) indicates the interval of overlap between *Df(2R)BSC273* and *Df(2R)BSC274* , both of which fail to complement *I.3.2* . This region includes thirteen protein-coding genes (blue bars), one of which is *cid* (red arrow). F) Alignment of the wild-type and *I.3.2 cid* protein coding nucleotide sequences (first 150 nucleotides only), highlighting the predicted single nucleotide deletion (136delA) in *I.3.2* (red). G) Alignment of the wild-type and *I.3.2* amino acid sequences, showing the truncation of CID in the *I.3.2* mutant well before the CID DNA binding domain (aa 119-223, gray region; SMART/EMBL). H) DNA sequencing chromatograms demonstrating the *cid* gene single nucleotide deletion in *I.3.2* . Coding DNA control sequence (upper panel) from *CyO* homozygous embryos, which matches both the FlyBase reference sequence and the DNA sequence from *Dark ^82[w+]^* / *CyO* control flies (as described in the text). In comparison, coding DNA sequences from *I.3.2* / *CyO* heterozygous adults (middle panel) and *I.3.2* homozygous embryos (lower panel) indicate a deletion present in the *I.3.2* chromosome. Note that the identities of homozygous embryos ( *I.3.2* / *I.3.2* and *CyO* / *CyO* ) were inferred from the resultant DNA sequences. The relevant CAG nucleotide triplet is indicated in the control panel (C, gray highlight; A, red asterisk; G, blue highlight), with the A (136) predicted to be deleted in *I.3.2* (Poly Peak Parser; Hill *et al.* 2014). This A peak becomes an A/C doublet in the *I.3.2* / *CyO* heterozygote DNA sequence (middle, though the doublet is called an A because of slightly higher fluorescence), and many double fluorescence peaks continue to the left (arrow) but not to the right (from G, blue, and onward). This is pattern is consistent with deletion of the A prior to the G on the coding strand (when sequencing the template strand/reverse reaction). This is confirmed in *I.3.2* homozygotes (lower panel) where sequencing shows the A nucleotide peak is missing and the flanking C (gray) and G (blue) nucleotides are directly adjacent, and that flanking nucleotides are all in register

## Description

The mutation *I.3.2* was previously isolated from a genetic screen for genes on chromosome arm 2R that regulate cell growth in a genetic background that blocks apoptosis (Kagey *et al.* 2012). Here we describe the effort of undergraduate researchers in the phenotypic analysis and genetic mapping of *I.3.2* . These groups of undergraduates, working independently at two different institutions, participated in courses implementing the Fly-CURE pedagogy, which teaches genetics concepts through inquiry-based learning in a course-based undergraduate research experience (CURE) (Bieser *et al.* 2018; Mast *et al.* 2022).

The effect of *I.3.2* on tissue growth was evaluated using the FLP/FRT recombination system (Xu and Rubin 1993) to generate homozygous mutant cells in the developing fly eye. The *I.3.2* mutation was generated by EMS mutagenesis of an *FRT42D* chromosome carrying *Dark ^82^* , a loss-of-function allele that blocks most apoptotic cell death in flies (Mills *et al.* 2006; Akdemir *et al.* 2006; Kagey *et al.* 2012). The *Dark ^82^* allele is included in the genetic background to facilitate identification of conditional growth mutations affecting cell growth and/or proliferation that may also, consequently, induce apoptosis. This approach has previously identified such mutations, and, importantly, *Dark ^82^* -mutant cell clones do not drastically impact eye development (Kagey *et al.* 2012; Stamm *et al.* 2019; Bieser *et al.* 2019; Talley *et al.* 2021). Because the *Dark ^82^* mutation is an insertional allele carrying a “mini- *white* ” transgene ( *w ^+mC^* ; Akdemir *et al.* 2006), mutant cell clones in the adult eye, for both control ( *Dark ^82^* ) and experimental ( *I.3.2* , *Dark ^82^* ) genetic backgrounds, are identified by the presence of red pigmentation when generated in a *white* background.

Flies heterozygous for *I.3.2* ( *w* ; *FRT42D I.3.2, Dark ^82^ /CyO* ) were crossed to flies carrying *eyeless-FLP* and an otherwise wild-type *FRT42D* chromosome ( *w* ; *FRT42D* ; *ey-FLP* ), and progeny flies with mosaic eyes (non- *Cy* ) were evaluated by microscopy. Adult eyes mosaic for control clones ( *Dark ^82^* only) show that mutant tissue composes approximately half of the eye with overall normal patterning (Fig. 1A), consistent with results previously reported (Kagey *et al.* 2012). By contrast, flies creating clones of *I.3.2* ( *I.3.2* and *Dark ^82^* together) fail to eclose, dying as late-stage pupae. Dissection of these dead pharate adults reveals a virtually “headless” phenotype (Fig. 1B). The eyes and most associated head structures are missing bilaterally in these individuals, with the common exception of some residual tissue that develops at the anterior midline, extending forward and down into what appears to be a proboscis-like structure (Fig. 1B-D). This phenotype appears to have complete penetrance as adult ‘escapers’ have not been observed.

To identify the gene associated with the *I.3.2* mutation, students began by crossing *I.3.2* to chromosomal deficiency stocks spanning 2R ( *Df* / *Cy* balancer, from the BDSC Deficiency Kit; Cook *et al.* 2012). Scoring for the presence or absence of non- *Cy* progeny ( *I.3.2* / *Df* flies *)* , students identified five non-complementing deficiencies (see Table 1), two of which ( *Df(2R)BSC331* and *Df(2R)ED2747* ) coincide with the position of *Dark* ( *Dark ^82^* is recessive-lethal). The other three deficiencies consisted of a single larger deficiency ( *Df(2R)CX1* ) and two smaller, overlapping deficiencies ( *Df(2R)BSC273* and *Df(2R)BSC274* ) with distal boundaries contained within the breakpoints of *Df(2R)CX1* (Fig. 1E, Table 1). The failure to complement *I.3.2* by both smaller deficiencies pointed to a location within the chromosomal region in common (2R: 13,430,464.. 13,502,150), which contained thirteen predicted protein coding genes (Fig. 1E). Students performed a second round of *I.3.2* complementation testing using lethal alleles for seven of the thirteen candidate genes (those available at the BDSC; Table 1) and identified a single non-complementing allele of the gene *centromere identifier* ( *cid* ), *cid ^T11-2^* (Blower *et al.* 2006). Two additional lethal *cid* alleles ( *cid ^T21-3^* and *cid ^G5950^* ; Blower *et al.* 2006; Raychaudhuri *et al.* 2012) also failed to complement the *I.3.2* mutation in subsequent analysis. Taken together, these results indicated that the *I.3.2* mutation likely resides in the *cid* gene and, accordingly, we have named this allele *cid ^I.3.2^* .

To determine whether the *I.3.2* mutation affected the *cid* protein coding region, students isolated genomic DNA from *I.3.2* heterozygote ( *w* ; *FRT42D cid ^I.3.2^ , Dark ^82^ /CyO* ) and control ( *w* ; *FRT42D Dark ^82^ /CyO* ) flies for PCR amplification and Sanger-based DNA sequencing. Chromatograms were analyzed using SnapGene Viewer that, in comparison to the *cid* reference sequence (FlyBase), identified several single nucleotide polymorphisms (SNPs) within *cid* in the *I.3.2* background. These SNPs were silent mutations and were also present in the control background (from which *I.3.2* was produced), and thus unlikely to represent *I.3.2* . However, in contrast to these shared SNPs, a difference in the *cid* DNA sequence was found in the region immediately beyond coding nucleotide 135. In the *I.3.2* / *CyO* background, sequence from nucleotide position 136 onward exhibited more than one fluorescence peak at each position, whereas this same region in the control background produced the expected single fluorescence peak at each position (Fig. 1H, upper and middle panels). These results were confirmed for both *I.3.2* and control DNA with multiple sequencing reactions over both strands.

Observing a long stretch of DNA sequence exhibiting multiple fluorescence peaks at each position is consistent with the presence of an insertion/deletion mutation (indel) on one chromosome. Sequencing such heterozygotes creates a “phasing” problem that affects all downstream chromatogram base calls because the indel and normal chromosome sequences are no longer in register. Thus, the *I.3.2* sequencing results were analyzed further using Poly Peak Parser (Hill *et al.* 2014), an online tool created specifically for the purpose of predicting indels in DNA sequences with an apparent phasing problem. Indeed, the *I.3.2* *cid* sequence was predicted to have a single nucleotide deletion that removes an adenine (A) nucleotide at position 136 (136delA) of the 678-nucleotide coding region (Fig. 1F). This deletion mutation creates a frameshift within codon 46 that changes two amino acids before creating a stop codon that truncates the protein (Fig. 1G). This predicted single nucleotide deletion was subsequently verified by sequencing DNA obtained from *I.3.2* homozygous embryos (Fig. 1H, lower panel).

The *centromere identifier* ( *cid* ) gene is a *Centromere protein A* ( *CENP-A* ) homolog encoding a histone H3 variant that incorporates into and marks centromeric chromatin (Blower and Karpen 2001; Blower *et al.* 2002, 2006). In the absence of CID, kinetochore assembly at the centromere is disrupted and a number of different kinetochore proteins mislocalize to other areas within the nucleus (Blower *et al.* 2006; Heun *et al.* 2006). One of these mislocalized proteins is the kinetochore kinase BubRI, which is known to activate the spindle assembly checkpoint (SAC). Loss of CID function causes cell cycle arrest, however this can be suppressed by concomitant loss of BubRI (Blower *et al.* 2006). This finding suggests that the SAC is sensitive to CID-mediated kinetochore assembly and can block cell cycle progression when CID function is perturbed. In the context of development, *cid* is an essential gene because loss-of-function mutants die as late-stage embryos after depletion of maternal CID from cellular pools (Blower *et al.* 2006).

Here we show that generating loss-of-function in *cid ^I.3.2^* cell clones within the developing eye causes a “headless” phenotype, where dead, pharate adults appear devoid of any cells derived from the eye-antennal imaginal disc. The only residual tissue developing anterior to the thorax appears similar to adult mouthparts, a point consistent with their development from separate labial and clypeolabral imaginal discs. This phenotype is similar to headless phenotypes previously observed with loss-of-function of the *eyeless* ( *ey* ) and *twin of eyeless* ( *toy* ) genes (Jiao *et al.* 2001; Kronhamn *et al.* 2002), and the Chaperonin containing TCP-1 (CCT) complex (Kim and Choi 2019). In these cases, the headless phenotype seems to be caused by early cell cycle arrest and subsequent activation of apoptosis. One important difference, however, in generating these headless phenotypes is that loss of *ey* , *toy* , or *CCT* function occurred throughout eye-antennal imaginal disc, whereas loss of *cid* ( *cid ^I.3.2^* ) occurred only within mitotic cell clones. It is plausible that loss of CID in such clones causes an autonomous block in the cell cycle and, perhaps, independent of Dark function, the activation of apoptosis. Why this would cause a headless phenotype is unclear, although it may be that arrested or dying *cid ^I.3.2^* -mutant cells ectopically drive the developmental arrest or death of wild-type sister cell clones. The phenomenon of non-autonomous cell “killing” (compensatory apoptosis) by autonomously dying cells has been described and is mediated by Tumor necrosis factor/Jun kinase signaling (Eiger/JNK in flies; Pérez-Garijo *et al.* 2013), though other mechanisms may exist. In the future, it will be worthwhile to examine the impact of mitotic *cid ^I.3.2^* cell clones on developing imaginal discs in larvae, in particular the relationship between cell genotype and cell death and the role of Eiger/JNK signaling in this process.

&nbsp;

&nbsp;

&nbsp;

**Table 1: Complementation tests conducted with mutant *I.3.2***

&nbsp;

**Table:** 

**Bloomington 2R Deficiencies Failing to Complement**			
**Deficiency**	**BDSC Stock #**	**Region**	** Deletes *Dark* ? **
*Df(2R)CX1/SM1*	442	2R: 12,700,421..14,062,629	No
*Df(2R)BSC273/CyO*	23169	2R: 13,159,579..13,502,150	No
*Df(2R)BSC274/CyO*	23170	2R: 13,430,464..13,593,272	No
*Df(2R)BSC331/CyO*	24356	2R: 16,869,330..17,139,923	Yes
*Df(2R)ED2747/SM6a*	9278	2R: 16,829,073..17,097,303	Yes
** Tested Genes Located Within the *Df(2R)BSC273/Df(2R)BSC274*** **Overlapping Region**			
**Gene**	**BDSC Stock #**	**Allele**	**Complementation Result**
*arr*	665	* arr ^k08131^*	Complements
&nbsp;	37088	* arr ^MI03803^*	Complements
&nbsp;	37186	* arr ^MI03378^*	Complements
&nbsp;	43026	* arr ^MI05833^*	Complements
*ATP8A*	81195	* ATP8A ^CR01153-TG4.1^*	Complements
*bbc*	28459	* bbc ^G3627^*	Complements
*cbc*	27620	* cbc ^T7-1^*	Complements
*cid*	27628	* cid ^T11-2^*	Fails to complement
&nbsp;	27629	* cid ^T21-3^*	Fails to complement
&nbsp;	29695	* cid ^G5950^*	Fails to complement
*cnn*	44788	* cnn ^MI08536^*	Complements
*drk*	12378	* drk ^10626^*	Complements
&nbsp;	56333	* drk ^MI11538^*	Complements

&nbsp;

## Reagents

*w; FRT42D, Dark ^82^ /CyO* (Akdemir et al., 2006)

*w; FRT42D, cid ^I.3.2^ , Dark ^82^ /CyO* (this manuscript)

*y w; FRT42D; ey-FLP* (BDSC 8211)

Bloomington *Drosophila* Stock Center 2R Deficiency Kit (Cook et al., 2012)

*cid ^T11-2^* / *CyO* (BDSC 27628)

*cid ^T21-3^* / *CyO* (BDSC 27629)

*cid ^G5950^* / *CyO* (BDSC 29695)

*Additional Bloomington Stocks (See Table 1 for complete list of stock numbers)*
